# Predictors of knowledge and adherence to COVID-19 safety protocols among nurses at health facilities in Tamale Metropolis of Northern Ghana

**DOI:** 10.1371/journal.pone.0274049

**Published:** 2022-09-06

**Authors:** David Abatanie Kanligi, Michael Boah, Martin Nyaaba Adokiya

**Affiliations:** 1 Department of Social and Behavioral Change, School of Public Health, University for Development Studies, Tamale, Ghana; 2 Pediatric Unit, Savelugu Municipal Hospital, Ghana Health Service, Northern Region, Ghana; 3 Department of Epidemiology, Biostatistics and Disease Control, School of Public Health, University for Development Studies, Tamale, Ghana; Georgia Southern University, UNITED STATES

## Abstract

**Background:**

Corona Virus Disease of 2019 (COVID-19) emerged in 2019 and caused widespread disruption to many facets of life, including healthcare. Healthcare workers, particularly nurses, became the front-line fighters against the pandemic, making it imperative to comply with recommended safety protocols. However, many nurses were infected by the virus in the Tamale Metropolis, raising concerns regarding their level of adherence to the safety protocols. This study assessed the predictors of knowledge and adherence to COVID-19 safety protocols among nurses at selected health facilities in the Tamale Metropolis of northern Ghana.

**Methods:**

A facility based cross-sectional study design was adopted and 339 nurses from six (6) public health facilities in the Tamale Metropolis were recruited for the study using questionnaires. The questionnaires were transformed into Google Forms for respondents to answer online via WhatsApp or email. The data were exported from the Google spreadsheet into SPSS and analyzed using descriptive and inferential statistics.

**Results:**

Of the 339 participants, 60.2% were classified as having adequate knowledge while only 9.1% demonstrated high adherence to COVID-19 safety protocols. Knowledge of COVID-19 was predicted by source of information, and marital status, whereas health facility types predicted level of adherence. The odds of having adequate knowledge were higher among unmarried nurses **(**AOR = 1.94; 95% CI: 1.16–3.25; p = 0.012**)** and nurses using social media (AOR = 1.80; 95%CI 1.02–3.18; p = 0.042) compared to their counterparts. Meanwhile, primary health care nurses (AOR = 0.24; 95% CI = 0.12–0.50; p<0.001) and secondary health care nurses (AOR = 0.52; 95% CI = 0.31–0.88; P = 0.016) had reduced odds of exhibiting higher adherence compared to nurses from tertiary-level facility.

**Conclusion:**

In this study, we found that knowledge was high but adherence to COVID-19 safety protocols was low. We suggest that facility managers should enforce compliance of their staff to the safety protocols to prevent spread of the virus within healthcare settings.

## Introduction

On the 30^th^ of January 2020, the World Health Organization (WHO) described COVID-19 as a condition of germane public health emergency and later declared it a pandemic on the 11^th^ of March, 2020 [1,2]. This resulted in major lockdowns and movement restrictions in many countries. The consequences included significant disruptions to socioeconomic development, stress, and the over burdening of nations’ health systems. Every nation experienced or is still experiencing the impact of COVID-19 as health workers became frontliners in the fight against the virus. This required behavioral modification in patient care [3]. Infection of health workers and caregivers, remain a major threat to COVID-19 control. Many health workers, including nurses and doctors, have contracted the virus [4]. An increasing spread among health workers, particularly clinical staff, undermines global efforts to controlling the pandemic. Though underreported infections have been cited, about 90,000 of all global cases were contributed by health workers as at August 2020 [5,6]. Similarly, over 10,000 infections have been reported among health workers in Africa. In some countries, health workers alone contribute about 10% of the disease prevalence [5].

COVID-19 infections among health workers in Ghana follow similar pattern as in sub-Saharan Africa and worldwide. It is estimated that over 2,000 health workers including nurses and doctors, have been infected, with the majority (>70%) being recorded in the southern part of Ghana [7–9]. As at July 2020, 32 health workers had been infected in Tamale Metropolis in the north of the country, with nurses comprising 37.5% infected individuals [10]. On the 10^th^ of August 2020, reports from the Northern Regional Health Directorate revealed that 145 health workers in the Metropolis had contracted the virus. These high infection rates among staff raise questions about the capacity of health workers, particularly nurses, to effectively implement COVID-19 infection prevention and control (IPC) measures.

The increasing COVID-19 infections among health workers have been attributed to knowledge, training, and adherence to the safety protocols [11]. This implies that health workers’ limited knowledge about the safety protocols may lead to poor adherence and increased exposure. Nurses constitute majority of the interdisciplinary health care team and have direct and continuous contact with the patient [12]. Thus, a focus on nurses would help contribute to our understanding of COVID-19 safety protocols implementation within the health care setting. Hence, this study assessed the factors predicting knowledge of and adherence to COVID-19 safety protocols among nurses at health facilities in the Tamale Metropolis.

## Materials and methods

### Study design and setting

A facility-based cross-sectional study was conducted among nurses at six public health facilities in the Tamale Metropolis, Ghana. The Metropolis is one of the six Metropolitan Assemblies in Ghana with an estimated land area of 646.90180 sq km [13]. It has three public hospitals, five health centers, a Christian Health Association of Ghana (CHAG) facility and over 20 private clinics and pharmacies [14].

Registered general nurses (RGNs) and enrolled nurses (ENs) were recruited for the study. These categories constitute the largest workforce providing acute care in the Ghanaian context and globally [12,15]. They are also, in most cases, the first contacts for patient care in established health facilities.

### Sample size calculation and sampling techniques

Nurses working in six public health facilities (including three hospitals and three health centers) were recruited for the study. The total number of nurses from these facilities at the time of the study was 1,736. This information was obtained from the human resource departments of the respective facilities. The sample size for the study was determined using Yamane’s formula for calculating sample size from a population of known size [16].

$$n=N/[1+N{\left(e\right)}^{2}]$$

Where; *n* is the sample size required for the study, *N* is the population size of the study setting (1736), and *e* is the level of desired precision (0.05) at a 95% confidence level. Thus, a minimal sample size of 325 was determined. A 10% non-response rate was added, and a total sample of 358 nurses was targeted.

A purposive sampling technique was used to select the three public health facilities in the Metropolis. Selecting the three hospitals was conceptually appropriate for the study because they are the only hospitals in the Metropolis and most of the nurses within the Tamale Metropolitan area are employed within these facilities [14]. However, the selection of the health centers was done through a simple random sampling technique using balloting. The names of all five health centers within the Tamale Metropolis (Nyohini, Bilipelaa, Moshie Zongo, Reproductive and Child Health (RCH) and Vittin) were written on pieces of paper. The papers were wrapped and mixed thoroughly on a plate. Three volunteers were asked to pick three papers out of the five which resulted in Nyohini, Moshie Zongo and Vittin being selected for the study. A census method (surveying all nurses who met the inclusion criteria) was employed to recruit nurses from the three health centers due to their small population sizes (total number of nurses from the three health centers = 101). The census method is appropriate for populations with smaller sample sizes as it eliminates errors associated with sampling procedures [16]. The remaining respondents were then distributed among the three hospitals using quota sampling. The quotas were determined by dividing the number of staff of each hospital by the combined total of all three hospitals (1635) and multiplying the resultant figure by the remaining sample size. Represented mathematically as: sample size of facility A = (A/A+B+C) X remaining sample size (remaining sample = 358–101 = 257). Details of the sampling are attached as (S1 Table).

At each of the three hospitals, a systematic random sampling approach was applied to select the respondents from each ward. The number of nurses in each ward was first collated. This information was then used to calculate quotas for the wards by dividing the total number of nurses in each ward by the number of nurses in the hospital and multiplying the results by the quota for that particular hospital. This is illustrated mathematically as; quota for a ward = (number of nurses in the ward)/ (total number of nurses in the hospital) X (the quota for that hospital). The resulting estimates became the number of nurses (quotas) sampled from the wards. Based on the quotas, sampling intervals were calculated using each ward’s register. The names in the register were arranged with serial numbers. At each of the wards, a name was randomly selected from the first five names as the random starting point. The sampling interval is then used to recruit the subsequent respondents until the total number of respondents required for the ward is achieved. In the event that a respondent declined to participate or did not meet the inclusion criteria, the slot automatically fell to the next name on the register. This was repeated until all respondents were recruited.

### Inclusion and exclusion criteria

Only registered general nurses and enrolled nurses were recruited for the study as they are mostly engaged in the provision of acute care services [15]. Specialist nurses, including midwives and psychiatry nurses, student nurses and nurses on rotation were excluded from the study.

### Data collection tool and procedure

A structured questionnaire was used to collect the data. The questions were adopted and modified from similar studies [17–25]. In addition, the WHO’s and Ghana’s Ministry of Health guidelines on COVID-19 infection prevention and control (IPC) were reviewed and used to design the questionnaire [26,27]. The questionnaire had three sections: socio-demographic characteristics, knowledge, and adherence of respondents. The questionnaire was then transformed into a Google Form with the ‘invite link’: https://docs.google.com/forms/d/1DAWhb-pk5i7u5NO-rtK_rjRK_YK1- rX9gN93wdta_t4/edit?vc = 0&c = 0&w = 1&flr = 0 and could only be accessed online. The aim was to limit personal contact and thus, reduced exposure of both researchers and the nurses.

All prospective respondents were approached and the nature of the study was explained to them. Those who consented to participate were sent the ‘invite link’ via WhatsApp/Gmail. Through the link, the respondents got access to the questionnaire, which they answered and submitted online. Before implementing the field survey, a pre-test study was conducted among 21 nurses in Tamale Seventh Day Adventist (SDA) Hospital and Tamale Reproductive & Child Health Centre. This provided information on the internal consistency and coherence of the data collection tool. A few modifications (including restructuring and rearranging of some questions) were made after the pre-test study. The study was conducted from 19^th^ January to 26^th^ February 2021. Data from the pre-test study were not included in the final analysis.

### Measurement of study variables

The dependent variables in this study are level of knowledge and adherence to COVID-19 safety protocols. The independent variables comprise socio-demographic characteristics of the respondents (sex, age, marital status and educational status) and healthcare factors (ward, cadre, roles, length of professional experience) and source of COVID-19 related information. These variables were measured using a series of questions adapted from similar studies [17–25].

Respondents’ knowledge regarding COVID-19 safety protocols was assessed using seven questions covering general knowledge of COVID-19 prevention. They had multiple choice responses. All questions were positively worded, and a score of 1 point was awarded for any option chosen, and a score of 0 point was awarded if the respondents failed to choose any of the options. The accumulative knowledge score ranged from 0 to 27 points. Using Bloom’s cut-off point as the reference, the overall knowledge of respondents was categorized as inadequate if they scored below 60% (less than 16 points), moderate if they scored 60–79% (16–21 points) and adequate if they scored 80–100% (22–27 points) as applied in previous studies [28–30].

The adherence level of respondents was measured in terms of how often they performed certain safety practices. A total of 11 questions were used to assess COVID-19 safety- related practices. Responses were graded on a 5-point Likert scale, which recorded the frequency of practice from ‘never’ to ‘always’. ‘Never’ was awarded 1 point, and ‘always’ 5 points. Negatively worded questions were inverted, in which case ‘never’ was awarded 5 points and ‘always’ was given 1 point. The maximum attainable score for the 11 questions was 55 points. Using Bloom’s cut off point as the basis [28,29], respondents were categorized as having low adherence if they scored below 60% (less than 33 points), fair adherence if they scored between 60–79% (33–44 points), and high adherence if they scored between 80–100% (44–55 points).

### Data processing and statistical analysis

Data from the online questionnaire was automatically collected in a Google spreadsheet, downloaded and exported to excel. The data was cleaned manually and exported into Statistical Package for Social Sciences (SPSS, version 25.0) for analysis. The field survey targeted 358 respondents. However, 354 (98.9%) completed the questionnaire. After cleaning, and excluding questionnaires with incomplete responses, 339 (94.7%) of them were used in the analysis.

After correcting and re-categorizing some variables, the data were analyzed and the results displayed using descriptive statistics. Furthermore, the association between each of the outcome variables and the predictor variables was assessed using ordinal logistic regression. The level of knowledge of the respondents was categorized as inadequate, moderate, or adequate, whereas the level of adherence was grouped into low, fair, and high. These were then modelled with socio-demographic factors as predictor variables. A p-value of less than 0.05 was considered statistically significant.

### Ethics consideration

Ethics approval for this study was granted by the Kwame Nkrumah University of Science and Technology (KNUST) Ethics Board and the Ghana Health Service Ethics Review Committee with review numbers CHRPE/AP/493/20 and GHS-ERC 026/02/21 respectively. In addition, an introductory letter from the Northern Regional Health Directorate was presented to each facility. The researchers explained the purpose and nature of the study to each respondent, including the potential risks and benefits of participating. Respondents were informed about their right to withdraw at any point during the study. They were also offered the opportunity to ask questions or seek clarifications before signing a written informed consent form. The anonymity and confidentiality of the respondents were ensured by removing personal identifiers and keeping the information collected private from any person outside the research team.

## Results

### Socio-demographic characteristics of respondents

A total of 339 (94.7%) of the respondents’ data were used in the analysis. The mean age of respondents was 29.3±4.2 years, and about half (49.9%) were between 26 and 30 years. More than half (56.0%) of the respondents were males, and 54.9% were married. About three-quarters (72.6%) of the respondents were engaged in the delivery of in-patient care services. Similarly, more than half (60.0%) of the respondents had less than four years’ professional experience as nurses. In addition, mass media (32.2%) and social media (27.4%) were the most frequently accessed sources of information for COVID-19 related updates (Table 1).

**Table 1:** Socio-demographic characteristics of nurses included in the study (n = 339).

Variable	Frequency	Percentage (%)
**Age (years)**		
<25	61	17.9
26–30	169	49.9
31–35	83	24.5
≥36	26	7.7
**Sex**		
Female	149	44.0
Male	190	56.0
**Marital status**		
Married	186	54.9
Single	150	44.2
Divorced	3	0.9
**Level of health facility**		
Primary	63	18.6
Secondary	128	37.8
Tertiary	148	43.7
**Educational qualification**		
Certificate	91	26.8
Diploma	146	43.1
Degree	96	28.3
Post graduate (Masters)	6	1.8
**Professional category**		
Enrolled nurse	105	31.0
Staff/senior nurse	148	43.7
Nursing officer/senior nursing officer	85	25.0
Principal nursing officer	1	0.3
**Area of work**		
Out-patient care	93	27.4
In-patient care	246	72.6
**Specific role at work**		
Clinical nursing	272	80.2
Non-clinical nursing	67	19.8
**Length of service experience**		
1–3	203	59.9
4–6	71	20.9
7–9	41	12.1
≥10	24	7.1
**Main source of COVID-19 information**		
Mass media (radio or TV)	109	32.2
Social media (WhatsApp, Facebook)	93	27.4
Ministry of health website	59	17.4
WHO website	40	11.7
Health facility management	35	10.4
Others (example, peers)	3	0.9

### Knowledge and adherence by nurses to COVID-19 safety protocols

**Fig 1:**
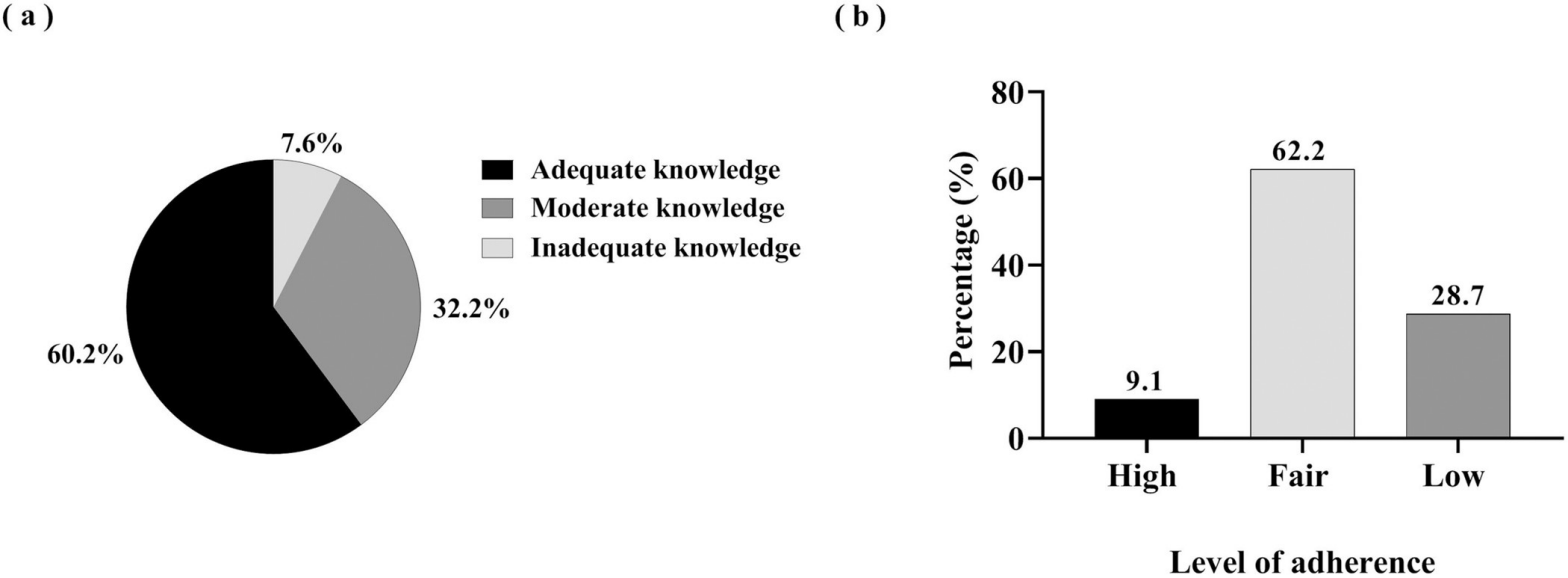
Level of: (a) knowledge about COVID-19 and (b) adherence to COVID-19 safety protocols among respondents (N = 339).

The majority (60.2%) of respondents had adequate knowledge of COVID-19 safety protocols (Fig 1A). Most (90%) of the respondents knew of fever and shortness of breath as typical features of COVID-19. Nearly all respondents (99.1%) identified the nose as the portal of entry for the coronavirus, followed by the mouth (78.5%) and the eye (64.0%). However, the most frequently mentioned preventive measures were wearing of a face mask (98.2%) and regular hand hygiene (93.5%). About 80% of the respondents also knew that patients must wear face masks when they are reporting to the health facility, coughing, sneezing, or in close contact with the staff. However, with the exception of alcohol, less than half of the respondents could identify sodium hypochlorate (34.8%) and povidone iodine (14.5%) as active substances against coronavirus. Details of the knowledge related variables are attached as (S2 Table).

However, less than 10% of the respondents scored high adherence to the safety protocols (Fig 1B). The data revealed that 53.2% and 65.7% of nurses washed their hands before and after patient contact, respectively. Similarly, the majority (72.2%) of the respondents reported sanitizing their hands after touching the patient’s surroundings. Less than a quarter (23.0%) of the respondents often use N95 respirators while 46.9% often use medical facemasks, and 38.9% reported using cloth facemasks during work. However, only 14.7% of the respondents reported being assisted to wear or take off their personal protective equipment (S3 Table).

### Factors associated with nurses’ knowledge

The proportional odds model on knowledge shows statistically significant improvement of the final model over the intercept only model with a p-value less than 0.05 (p<0.001); and corresponding non-significant values for the goodness of fit measures (Pearson Chi-square; p = 0.495 and Deviance Chi-square; p = 0.850) which gives an indication of good model fit. Also, test of parallel line was non-significant (p = 0.138) implying the data met the proportional odds assumption.

The model indicated that knowledge of respondents was predicted by marital status and source of COVID-19 related information. The odds of having adequate knowledge (versus moderate or poor knowledge) were higher among unmarried nurses compared to married nurses **(**AOR = 1.94; 95% CI: 1.16–3.25; p = 0.012**)**. Also, the odds of having adequate knowledge (versus moderate or poor knowledge) were higher among nurses using social media compared to nurses who accessed COVID-19 related information from websites or facility management (AOR = 1.80; 95%CI 1.02–3.18; p = 0.042). Furthermore, the odds off having adequate knowledge were almost two times among mass media users compared to those who depended on websites or facility management for COVID-19 related updates (Table 2).

**Table 2:** Factors associated with nurses’ knowledge of COVID-19 safety protocols at health facilities in Tamale Metropolis, Ghana (n = 339).

Variable	AOR (95% CI)	p-value
**Age (years)**		
<26	0.52(0.16–1.64)	0.263
26–30	1.35(0.48–3.80)	0.573
31–35	2.32(0.90–5.96)	0.081
≥35 (Reference)		
**Sex**		
Male	0.72 (0.45–1.15)	0.169
Female (Reference)		
**Marital status**		
**Single**	**1.94(1.16–3.25)**	**0.012**
Married (Reference)		
**Educational Level**		
Diploma holders	0.85(0.43–1.69)	0.643
Degree/masters	1.52(0.85–2.71)	0.159
Certificate (Reference)		
**Specific role**		
Non-clinical nurses	0.89 (0.51–1.52)	0.662
Clinical nurses (Reference)		
**Length of work**		
Above 6 years	1.70 (0.76–3.78)	0.196
Below 7 years (Reference)		
**Level of facility**		
Primary	0.76(0.37–1.55)	0.455
Secondary	0. 72 (0.41–124)	0.237
Tertiary (Reference)		
**Source of information**		
Social media	**1.80(1.02–3.18)**	**0.042**
Mass media	**1.74 (1.01–3.00)**	**0.044**
Websites/facility managements (Reference)		

### Factors associated with nurses’ adherence

The indices for the adherence model also suggested good model fit with a statistically significant value for the model fitting information (p = 0.026) and a corresponding non-significant value for the goodness-of-fit test (Pearson Chi-square; p = 0.163; and Deviance Chi-square; p = 0.956). The test of parallel lines produced a non-significant value (p = 0.166) which indicates that the data met the proportional odds assumption.

The level of healthcare facility showed statistically significant association with adherence to COVID-19 preventive protocols. The odds of having higher adherence (versus fair or low adherence) among nurses from primary healthcare facilities was 0.24 times lower compared to those from tertiary facilities (AOR = 0.24; 95% CI = 0.12–0.50; p<0.001). In addition, the odds of having higher adherence among nurses from secondary healthcare facilities was 0.52 lower compared with those from tertiary facilities (AOR = 0.52; 95% CI = 0.31–0.88; P = 0.016). Nurses from primary and secondary level facilities were likely to exhibit low adherence to the protocols compared with their colleagues from tertiary facilities (Table 3).

**Table 3:** Factors associated with nurses’ level of adherence to COVID-19 safety protocols at health facilities in Tamale Metropolis, Ghana (n = 339).

Variable	AOR (95% CI)	p-value
**Age (years)**		
<26	1.74(0.63–5.70)	0.357
26–30	1.78(0.62–5.12)	0.284
31–35	1.48 (0.57–3.88)	0.422
≥35 (Reference)		
**Sex**		
Male	1.08 (0.68–1.69)	0.750
Female (Reference)		
**Marital status**		
Single	1.50(0.91–2.48)	0.115
Married (Reference)		
**Educational Level**		
Diploma holders	1.46(0.74–2.91)	0.269
Degree/masters	1.12(0.64–1.97)	0.688
Certificate (Reference)		
**Specific role**		
Non-clinical nurses	0.89(0.51–1.54)	0.680
Clinical nurses (Reference)		
**Length of work**		
Above 6 years	1.23 (0.58–2.62)	0.590
Below 7 years (Reference)		
**Level of facility**		
Primary	**0.24(0.12–0.50)**	<**0.001**
Secondary	**0.52(0.31–0.88)**	**0.016**
Tertiary (Reference)		
**Source of information**		
Social media	0.81 (0.47–1.42)	0.467
Mass media	0.83(0.49–1.41)	0.489
Websites/facility managements (Reference)		

## Discussion

In this study, we assessed predictors of nurses’ knowledge and adherence to COVID-19 safety protocols at selected health facilities in the Tamale Metropolis of northern Ghana. More than half (60.2%) of nurses surveyed had adequate knowledge of COVID-19 safety protocols in terms of the route of infection, protective and preventive measures and virus deactivation substances. This finding is similar to previous findings. According to one study, 3 out of 5 (60%) healthcare providers had adequate knowledge of COVID-19 safety protocols [2]. In the current study setting, knowledge level may help to manage and control the pandemic effectively. There is evidence to suggest that knowledge of the disease serves as the foundation for taking preventive measures [31–33]. Besides, informed decisions are made on the premise of knowledge that individuals gain [32]. However, in China and Iran, some studies found that 9 out of 10 health workers were knowledgeable about COVID-19 safety protocols [34,35]. Thus, the level of reported knowledge on the clinical features, route of infection, and preventive measures of COVID-19 in the previous studies is higher than in the current setting. Ideally, knowledge of the pandemic should increase with the passage of time [36]. Reasons for this comparatively lower knowledge of nurses in Ghana may be due to the less severe nature of the pandemic in the country [37]. Typically, health workers would usually make efforts to learn about any emerging disease. The perceived severity on the other hand, influences the effort committed to such endeavors [38]. This could explain why health workers in China and Iran had a better understanding of COVID-19 prevention. Educational interventions are crucial for learning and may help improve healthcare workers’ knowledge of COVID-19 prevention strategies [39].

Our findings revealed that marital status was associated with nurses’ knowledge level. In addition, knowledge of nurses varied by the source of COVID-19 related updates. Nurses who were married and obtained information from mass/social media had greater odds of possessing adequate knowledge on COVID-19 prevention. This finding corroborates previous research that found social and mass media to be associated with healthcare providers’ knowledge [40]. The finding thus, support previous observation that single people spend more time on the internet than married people [41]. Unmarried nurses in our study might have spent more time on social media or internet during the pandemic and indirectly learnt more about preventive measures than their married counterparts. Therefore, married nurses may need more time to learn and cope with evolving health issues. It may be necessary to employ targeted training for married health workers regarding COVID-19 prevention.

In this study, we found that only 9.1% of nurses strictly adhered to COVID-19 safety protocols. This finding differs from other studies. A study conducted in the Offinso North District of Ashanti Region reported that more than half (58%) of the health workers reported higher adherence to COVID-19 safety protocols [2]. There is disparity in terms of adherence between northern and southern Ghana. This variation could be explained by the country’s different COVID-19 infection rates and patterns. More health workers (over 70%) in the southern part of Ghana were infected with the virus than in the northern part [9,42]. In addition, differences in safety protocols compliance within the same country setting may be due to the period in which the two studies were conducted. COVID-19 risk perception was high during the first wave of the pandemic [43]. Because of the fear of infection associated with the virus, health workers may have taken extra precautions. However, the apparent lack of seriousness attached to COVID-19 related issues currently may have resulted in apathy towards its safety protocols [44–46]. This may have resulted in low adherence among Ghanaians and more likely, among health workers. Strict adherence to the safety protocols is critical for preventing recurrent infections, virus mutations, and repeated waves of the pandemic.

Northern Ghana’s situation differs from that of other countries. More than half of the healthcare providers in Nigeria, Uganda and China; 57%, 74%, and 99%, respectively, complied strictly to COVID-19 safety protocols [29,47,48]. This variation of adherence among health workers across different geographical zones signifies the possible interplay of some influencing factors. There was adequate support system for COVID-19 prevention in China. Personal Protective Equipment (PPEs) in the form of gloves, face masks, N-95 respirators, overalls, boots and the like were provided in sufficient quantities whilst environmental control measures were effectively enforced [48]. Due to strict adherence to the safety measures, there were no infections among healthcare providers who attended to COVID-19 patients in China’s Hubei Region [48]. In Africa, support system in the form of personal protective equipment for health workers were generally lacking [5]. In addition, reports also suggest inadequate preparation for COVID-19 prevention in certain areas [20,49,50]. Thus, there is a need to intensify strict adherence to the safety protocols among health workers. This requires all health facilities to have an elaborate IPC program that would provide adequate support system for COVID-19 prevention [27].

The data from our study revealed that the adequate knowledge of nurses did not reflect their level of adherence to the safety protocols. Less than one-tenth of the respondents showed a high level of adherence to COVID-19 safety measures. In comparison to the more than 60% of respondents who had adequate knowledge, adherence to the safety protocols was woefully low. This finding contradicts the findings of previous studies, which found that having adequate knowledge resulted in higher adherence [51,52]. However, other studies suggested that adequate knowledge does not always result in higher adherence [53]. Knowledge alone may not be enough to influence compliance. Other factors related to the individual, facility, or even the guidelines influence how health workers adhere to the safety protocols [54,55]. Thus, there must be adequate support system to facilitate implementation of COVID-19 safety directives.

In addition, our data showed that nurses from primary or secondary healthcare facilities were more likely to have low adherence than those from tertiary care facilities. This is consistent with the findings of a study conducted in Nigeria where the type of health facility was associated with healthcare workers’ adherence [17]. When compared to primary or secondary healthcare institutions, tertiary healthcare facilities have more resources to implement infection prevention and control guidelines [56]. As a result, nurses in tertiary facilities may have the enabling environment to adhere to the COVID-19 safety protocols than their counterparts from primary or secondary facilities. Therefore, nurses from primary and secondary care facilities may need support, including logistics and regular training to improve their level of compliance with the safety protocols.

### Strengths and limitations

This study had strengths. The sample size was relatively large. Moreover, the health facilities were representative of the Ghana health system structure. This study also had some limitations. It is a cross-sectional study and cannot establish causality. Similarly, recall bias could have occurred due to memory loss. The level of knowledge and adherence were categorized into three responses, unlike other studies which used two categories. The comparatively lower levels observed could be due to this categorization. In addition, the study is geographically and contextually bound, and the findings may not be applicable to other settings and other categories of healthcare professionals. In the current study, adequate knowledge of respondents did not translate into high adherence. Future studies should focus on exploring why nurses with adequate knowledge do not exhibit high adherence to COVID-19 safety protocols.

### Conclusion

Although the majority of the nurses in this study reported adequate knowledge of COVID-19, only a few adhered to the recommended safety guidelines. The level of knowledge of respondents varied according to source of COVID-19 related information and marital status, whereas adherence was predicted by the type of health facility. We recommend that facility managers should enforce compliance of their nursing staff to the COVID-19 safety protocols to prevent spread of the virus within health care settings.

## Supporting information

S1 Table Study population and sample from six health facilities in Tamale Metropolis, Ghana. (PDF)

S2 Table Nurses’ knowledge of COVID-19 features and its preventive measures at health facilities in Tamale, Ghana (n = 339). (PDF)

S3 Table Nurses*’* adherence to COVID-19 safety protocols at health facilities in Tamale, Ghana (n *=* 339). (PDF)
