# Supplementary material for: Predictors of knowledge and adherence to COVID-19 safety protocols among nurses at health facilities in Tamale Metropolis of Northern Ghana
Source: PLoS One. 2022 Sep 6;17(9):e0274049. doi: 10.1371/journal.pone.0274049 (PMC9447918; doi:10.1371/journal.pone.0274049)
Supplement: S1 Table — (PDF) [file pone.0274049.s001.pdf]

S1 Table: Study population and sample from six health facilities in Tamale Metropolis, Ghana

Health facility	Number of nurses	Number sampled
Tamale Teaching Hospital	830	130
Tamale Central Hospital	430	66
Tamale West Hospital	385	61
Nyohini Health Centre	45	45
Vittin Health Centre	33	33
Moshie Zongo Health Centre	23	23
Total	1,736	358
