# Supplementary material for: Predictors of knowledge and adherence to COVID-19 safety protocols among nurses at health facilities in Tamale Metropolis of Northern Ghana
Source: PLoS One. 2022 Sep 6;17(9):e0274049. doi: 10.1371/journal.pone.0274049 (PMC9447918; doi:10.1371/journal.pone.0274049)
Supplement: S2 Table — (PDF) [file pone.0274049.s002.pdf]

S2 Table: Nurses' knowledge of COVID-19 features and its preventive measures at health facilities in Tamale, Ghana (n= 339)

Variable	Frequency	Percentage
**Typical features of COVID-19**		
Fever	313	92.3
Shortness of breath	309	91.2
Taste/smell lose	262	77.3
Dry cough	274	80.8
Fatigue	240	70.8
**COVID-19 route of infection**		
Eye	217	64.0
Mouth	266	78.5
Nose	336	99.1
**Self-protective measures during patient care**		
Observe social distancing	192	56.6
Wearing face Mask/Shield	333	98.2
Wearing gloves	302	89.1
Practicing regular hand hygiene	317	93.5
**Measures to prevent spread in health facility**		
Isolation of suspects	317	93.5
Restricting visitors	270	79.6
Patient spacing	292	86.1
Disinfecting regularly touch surfaces	295	87.0
**Substances that can deactivate coronavirus**		
70% Alcohol	316	93.2
0.5% Sodium Hypochlorate	118	34.8
1% Povidone Iodine	49	14.5
**Management of staff with COVID-19**		
S/he should be quarantined	326	96.2
S/he should be monitored for symptoms	285	84.1
S/he should be tested	314	92.6
S/he should be counselled	219	64.6
**Moments during which patients should mask up**		
Coughing	305	90.0
sneezing	295	87.0
in close contact with the nurse	281	82.9
coming into the facility	294	86.7
