# Supplementary material for: Predictors of knowledge and adherence to COVID-19 safety protocols among nurses at health facilities in Tamale Metropolis of Northern Ghana
Source: PLoS One. 2022 Sep 6;17(9):e0274049. doi: 10.1371/journal.pone.0274049 (PMC9447918; doi:10.1371/journal.pone.0274049)
Supplement: S3 Table — (PDF) [file pone.0274049.s003.pdf]

S3 Table: Nurses' adherence to COVID-19 safety protocols at health facilities in Tamale, Ghana (n= 339)

Variable	Response				
	Never n(%)	Occasionally n(%)	Sometimes n(%)	Often n(%)	Always n(%)
Frequency of using N95 respirator	103(30.4)	92(27.1)	67(19.8)	32(9.4)	45(13.3)
Frequency of using medical facemask	41(12.1)	64(18.9)	70(20.6)	75(22.1)	89(26.3)
Frequency of using cloth facemask	65(19.2)	74(21.8)	68(20.1)	58(17.1)	74(21.8)
Frequency of wearing facial protection	73(21.5)	152 (44.8)	58(17.1)	29(8.6)	27(8.0)
Frequency of performing hand hygiene before touching a patient/patient surrounding	7(2.1)	19(5.6)	43(12.7)	88(26.0)	182(53.7)
Frequency of performing hand hygiene after touching a patient or patient surrounding	4(1.2)	10(2.9)	29(8.6)	75(22.1)	221(65.2)
Frequency of sanitizing hands after touching surfaces in the hospital	6(1.8)	25(7.4)	63(18.6)	114(33.6)	131(38.6)
Frequency of a train observer assisting you to wear/take off PPEs	178(52.5)	62(18.3)	49(14.5)	37(10.9)	13(3.8)
Frequency of exceeding your usually duty schedule during this pandemic	38(11.2)	61(18.0)	98(28.9)	84(24.8)	58(17.1)
Frequency of wearing medical gloves when in direct contact with patient	28(8.3)	22(6.5)	74(21.8)	90(26.5)	125(36.9)
Frequency of disinfecting highly touched surfaces in your ward	12(3.5)	50(14.7)	87(25.7)	83(24.5)	107(31.6)
